# Association Between Lipids, Lipoproteins Composition of HDL Particles and Triglyceride-Rich Lipoproteins, and LCAT and CETP Activity in Post-renal Transplant Patients

**DOI:** 10.1007/s12013-013-9559-y

**Published:** 2013-03-12

**Authors:** Elżbieta Kimak, Jerzy Bylina, Janusz Solski, Magdalena Hałabiś, Iwona Baranowicz-Gąszczyk, Andrzej Książek

**Affiliations:** 1 Department of Laboratory Diagnostics, Medical University, Lublin, Poland; 2 Department of Informatics and Statistics, Institute of Rural Health, Lublin, Poland; 3 Department of Nephrology, Medical University, Lublin, Poland

**Keywords:** Apolipoprotein (apo)AI, apoB, apoAII, apoAIInonB, apoB-containing apoAII, apoCIII, apoCIIInonB, apoB-containing apoCIII, LCAT, CETP, hsCRP, Renal transplant

## Abstract

High-density lipoprotein (HDL) remodeling within the plasma compartment and the association between lecithin-cholesterol acyltransferase (LCAT) and cholesterol ester transfer protein (CETP) activity, and lipid, lipoprotein concentrations and composition were investigated. The aim was to examine the high sensitivity of C-reactive protein (hsCRP), lipid, apolipoprotein B (apoB), apoAI, total apoAII, apoAIInonB, apoB-containing apoAII (apoB:AII), total apoCIII, apoCIIInonB, apoB-containing apoCIII (apoB:CIII) concentration and LCAT and CETP activity to gain an insight into the association between them and LCAT and CETP, 57 post-renal transplant (Tx) patients with and without statin therapy and in 15 healthy subjects. Tx patients had moderate hypertriglyceridemia, hypercholesterolemia, and dyslipoproteinemia, disturbed triglyceride-rich lipoproteins (TRLs) and HDL composition, decreased LCAT, and slightly increased hsCRP but no CETP activity. Spearman’s correlation test showed the association between lipids and lipoproteins and LCAT or CETP, and multiple ridge stepwise forward regression showed that immunosuppressive therapy in Tx patients can disturb HDL and TRLs composition. The results suggest that inhibition or activation of LCAT is due, in part, to HDL-associated lipoprotein. Lipoprotein composition of apoAI, apoAIInonB, and apoCIIInonB in HDL particle and apoB:AII TRLs can contribute to decrease LCAT mass in Tx patients. Tx patients without statin and with lower triglycerides but higher HDL cholesterol concentration and disturbed lipoprotein composition of ApoAI and apoAII in HDL particle can decrease LCAT, increase LDL cholesterol, aggravate renal graft, and accelerate atherosclerosis and chronic heart diseases.

## Introduction

Lecithin-cholesterol acyltransferase (LCAT) is a plasma enzyme that esterifies cholesterol and raises high-density lipoprotein cholesterol (HDL-C). Cholesteryl esters (CE) are significantly more hydrophobic than free cholesterol and are formed by LCAT partition from the surface of lipoprotein to the hydrophobic core. This transforms the small pre-β HDL into larger, spherical-shaped α-migrating forms of high-density lipoprotein (HDL). The increase in size of the HDL stabilizes it from removal by renal clearance. LCAT prevents the reverse exchange of cholesterol by passive diffusion from HDL to the peripheral cells and also promotes net removal of cholesterol from the peripheral cells to the HDL [1, 2]. After cholesterol esterification by LCAT, the cholesteryl esters on the HDL are transferred to apoB-containing lipoproteins by cholesteryl ester transfer protein (CETP), although approximately 25 % of cholesteryl esters are directly formed on apoB-containing lipoproteins. The cholesteryl esters are delivered to the liver for excretion, either by the uptake of low-density lipoprotein (LDL) by the LDL receptor, or by selective lipid uptake by scavenger B receptor type I (SB-BI) in the liver [2–5]. LCAT is a key factor in promoting the formation of the HDL particle containing apoAI and apoAII by fusion of the spherical α-HDL-containing apoAI and nascent discoid HDL-containing apoAII. Distribution of HDL apolipoproteins, which are exchangeable, is modified by LCAT, CETP, phospholipid transfer protein (PLTP) and hepatic lipase. The HDL structural remodeling and apoAI dissociation are essential for reversing cholesterol transport (RCT) [2, 6]. LCAT is an enzyme in the HDL metabolism and RCT and may play an important role in modulating plasma triglycerides (TG) levels, but the underlying mechanism has not been explored nor has the association with apoCIII, apoCIIInonB, apoB-containing apoCIII (apoB:CIII); apoAII, apoAIInonB, apoB-containing apoAII (apoB:AII); and lipids [7]. Its role in human atherosclerosis in post-renal transplant patients (Tx) with chronic kidney disease has not been definitively established and is likely to be context dependent [8]. Studies have produced conflicting results for the anti- or pro-atherogenic role of LCAT [9–11]. Therefore, the exact role of LCAT in the pathogenesis of atherosclerosis is still not fully known. Unfortunately, there is no information about the association between LCAT and CETP activity, and the concentration and composition of lipoprotein HDL and triglyceride-rich lipoproteins (TRLs) metabolism in Tx patients without active inflammatory.

The aim of the present study was to examine hsCRP, lipids, apoB, apoAI, total apoAII, apoAIInonB, apoB:AII, total apoCIII, apoCIIInonB, apoB:CIII concentration, and LCAT and CETP activity to gain an insight into the association between them and LCAT and CETP in 57 Tx patients without acute inflammatory, but with and without statin therapy.

## Materials and Methods

### Patients Selection

A total of 57 post-renal transplant patients (Tx) (28 males and 29 females) aged 19–66 years and 15 apparently normolipidemic healthy individuals as controls were recruited for the study. The Tx patients had undergone treatment in the Nephrology Department of the Medical University in Lublin. These patients did not have proteinuria, active inflammatory disease, liver disease, malignancy or diabetes mellitus, but did have hypertension (*n* = 38) and cardiovascular disease (*n* = 1). The causes of kidney disease in the post-renal transplant patients were the following: glomerulonephritis in 48 patients, interstitial nephritis in 7, and unknown in 2. The Tx patients with hypertension were using anti-hypertensive medications of either calcium channel blockers or angiotensin-converting enzyme antagonists, angiotensin II receptor subtype 1 (AT1) blockers and α-blockers, but no diuretics were used in any of the studied groups. The number of hyperlipidemic patients treated with atorvastatin or simvastatin is 25 (*n* = 25), but 32 Tx patients did not receive statin therapy. All Tx patients on immunosuppressive treatment received calcineurin inhibitors and prednisone. Statin-treated patients received mainly cyclosporine (60 % of Tx patients). In this group, the Tx patients received CSA + Myfortic, CSA + Azatiopryne, and CSA + CellCept; the patients were given CSA, and Prograf + Myfortic and Prograf + CellCept and CellCept + Myfortic. Tx patients without satin therapy received mainly prograf (60 % of Tx patients) along with Myfortic, CellCept and CSA + Myfortic, together with CSA + Azatiopryne and CSA + CellCept. One person received only prednisone. All Tx patients were divided into three groups: all patients, those with statin therapy, and those without. In patients without statin group, those who had lower TG and higher HDL C concentrations were selected.

The study was conducted in accordance with the guidelines of the Ethics Committee of the Medical University in Lublin, Poland.

### Detection of Lipids, Lipoproteins, hsCRP, and Routine Laboratory Parameters

Lipids, lipoproteins, and routine laboratory parameters were obtained in serum after a 14-h overnight fasting. Blood was taken from vein and transferred to commercial test tubes. Serum was immediately separated and stored in aliquots at −80 °C until use. Using routine laboratory parameters, the level of creatinine and lipid was measured on a Siemens analyzer (Germany). Low-density lipoprotein cholesterol (LDL-C) was calculated according to the Friedewald’s formula [12]. Non-HDL-C was calculated as total cholesterol (TC) minus HDL-C. Lipoproteins apoAI, apoB, and high-sensitivity C-reactive protein (hsCRP) were determined with immunonephelometric methods, using the Health Care Diagnostic Product (Siemens GmbH, Germany) on a Dade Behring nephelometer BNII System (Germany). Triglyceride-rich lipoproteins (apoB:CIII and apoB:AII) were separated as non-HDL lipoproteins using anti-apoB antibodies of Tx patients and healthy subjects. Total apoCIII and apoCIIInonB were measured by electroimmunodiffusion, according to Laurell, using a commercial kit (Sebia, USA), and total apoAII and apoAIInonB were measured with immunonephelometric methods, using Siemens Health Care Diagnostic Product on a Dade Behring nephelometer BNII System. ApoB:AII was calculated as total apoAII minus apoAIInonB. ApoB:CIII was calculated from the difference between the total apoCIII and apoCIIInonB.

## Detection of LCAT and CETP Activity

A serum CETP assay kit (BioVision Inc., CA, USA), using a donor molecule containing a fluorescent self-quenched neutral lipid was transfererred to an acceptor molecule results in fluorescence (Excitation: 465 mn; Emission: 535 nm). Serum LCAT assay kit (ELISA, Life Inc., Wuhan, China) was used. The kit is a sandwich enzyme immunoassay for the in vitro quantitative measurement of human LCAT in serum. The microtiter plate provided in this kit had been pre-coated with an antibody specific to LCAT. Standards or samples are then added to the appropriate microtiter plate wells with a biotin-conjugated polyclonal antibody preparation specific for LCAT. Next, avidin in conjunction with horse radish peroxidase (HRP) was added to each well. Only those wells that contained LCAT, biotin-conjugated antibody and enzyme-conjugated avidin exhibited a change color. The enzyme-substrate reaction was terminated by the addition of a sulfuric acid, and the color change was measured spectrophotometrically at a wavelength of 450 nm ± 10 nm. The LCAT mass (U/L − 1 μmol/min/L = amount of enzyme that catalyzes the reaction of 1 μmol of substrate per minute under standard conditions) activity in the samples was then determined by comparing the O.D. of the samples to the standard curve.

### Statistical Analysis

The data were expressed as medians and minimum–maximum. Statistical analysis of the results obtained was performed using the nonparametric Kruskal–Wallis test for comparison of Tx patients groups and the controls. The variables with skewed distribution were log-10-transformed. However, the values in tables are presented as non-transformed data. The relation between CETP or LCAT activity and concentration of lipid, lipoprotein and lipid and lipoprotein ratios were examined by Spearman’s correlation analysis. Multiple ridge stepwise forward regression analysis was used to investigate the relationship between LCAT as dependent variable and lipids, apoAI, apoB, apoCIII, apoCIIInonB, B:CIII, apoAII, apoAIInonB, apoB:AII, hsCRP concentrations, and CETP activity as non-dependent variable. In the model of multiple regression analysis, high correlations between predictor variables result in inadequate regression coefficients. In such cases, multiple ridge stepwise forward regression analysis improves the accuracy of the model. In the model of multiple ridge forward stepwise regression analysis, LCAT was selected as the dependent variable and lipoproteins as the non-dependent variable, and for each of the non-dependent variables, parameters were calculated according to the equation: *y* = *β*_0_ + *β*_1_ *x*_1_ + *β*_2_ *x*_2_ + … + *β*_*n*_ *x*_*n*_. The relationship between the dependent variables is expressed by the coefficient of multiple regression (*β*), which provides information about the relationship between the dependent LCAT and the non-dependent variables. After adjustment for age and gender, multiple ridge stepwise forward regression analyses were performed in Tx patients. The statistical significance of all variables was established at *P* < 0.05, and statistical analysis was performed using the Statistica programme (StatSoft, Krakow, Poland).

## Results

Table 1 presents the results of the clinical and laboratory parameters of Tx patients and the reference group. Concentration of serum lipids (TG, TC, LDL-C, HDL-C, non-HDL-C), lipoproteins (apoAI, apoB, total apoAII, apoAIInonB, apoB:AII, total apoCIII, apoCIIInonB, apoB:CIII), hsCRP, LCAT and CETP activity, and lipid (TC/HDL-C, LDL-C/HDL-C, TG/HDL-C) and lipoprotein (HDL-C/apoAI, apoAI/apoB, apoAI/apoCIII) ratios in Tx patients with and without statin therapy is given in Table 2. The in vivo results of the present study show that Tx patients with and without statin therapy had moderate hypertriglyceridemia, hypercholesterolemia, dyslipoproteinemia, and atherogenic lipid and lipoprotein ratios, decreased LCAT mass, and slightly increased hsCRP, but no CETP activity. However, Tx patients with statin therapy had a higher concentration of LDL-C, apoB, and triglyceride-rich lipoproteins (TRLs-apoB:CIII, apoB:apoAII) and lipid ratios, but lower concentration of HDL-C and lipoprotein ratios than Tx patients without statin and the reference group.

**Table 1:** Clinical and routine laboratory parameters in the reference group and post-renal transplant patients (Tx)

	Reference group *(n* = 15)	Tx patients with statin *(n* = 25)	Tx patients without statin *(n* = 32)	All Tx patients *(n* = 57)
Age (years)	44 (22–50)	51 (20–66)	38 (19–61)	44 (19–66)
Gender (male/female)	7 M, 8F	13 M, 12F	15 M, 17F	28 M, 29F
BMI (kg/m^2^)	21.0 (18.5–24.7)*	25.6 (20.1–35.2)*	22.9 (20.4–27.4)	24.3 (19.9–35.2)*
eGFR M (mL/min/1.73 m^2^)	120 (103–127)	81 (60–131)	84 (62–143)	81 (60–143)
eGFR F (mL/min/1.73 m^2^)	108 (98–120)	68 (51–91)	71 (51–122)	69 (51–122)
Time after transplantation (years)	–	5.8 (1–15)	4.36 (1–13)	5.1 (1–15)
Creatinine (μmol/L)	74 (62–99)	106 (61.9–159.1)	106 (61.9–141.4)	106 (61.9–159.1)
Albumin (g/L)	44.1 (42.2–47.9)	43.0 (41.1–48.2)	43.7 (42.0–48.3)	43.2 (41.1–48.3)

*BMI* body mass index, *eGFR* estimated glomerular filtration rate, *M* male, *F* female

Values are expressed as median (min–max): * P < 0.05, ** P < 0.01 versus reference group

**Table 2:** Concentration of lipids, lipoproteins, lipid and lipoprotein ratios, hsCRP, LCAT and CETP activity in the reference group and in post-renal transplant patients (Tx) with and without statins

	Reference group *(n* = 15)	Tx patients with statin (*n* = 25)	Tx patients without statin (*n* = 32)	All Tx patients *(n* = 57)
TG (mmol/L)	0.98 (0.45–0.85)	1.39 (1.05–2.37)**	1.35 (0.73–1.88)**	1.38 (0.73–2.37)**
TC (mmol/L)	4.60 (3.55–4.82)	5.18 (3.42–6.16)	5.05 (3.60–5.90)	5.08 (3.42–6.16)
LDL-C (mmol/L)	2.22 (1.19–2.59)	3.37 (2.05–4.56)*	3.16 (1.35–4.45)	3.26 (1.35–4.56)
HDL-C (mmol/L)	1.79 (1.56–2.02)	1.11 (0.77–1.71)*	1.30 (0.90–1.93)	1.28 (0.77–1.93)
nHDL-C (mmol/L)	2.82 (1.56–2.02)	4.04 (2.64–5.30)	3.70 (2.43–4.92)	3.83 (2.83–5.30)
apoAI (mg/L)	1690 (1540–2660)	1570 (1010–2113)	1551 (1257–1973)	1560 (1001–2113)
apoB (mg/L)	690 (550–670)	989 (599–1558)*	832 (510–1151)	860 (510–1550)
apoAII (mg/L)	292 (246–354)	322 (179–498)	322 (243–436)	324 (179–498)
apoAIInB (mg/L)	288 (28.2–341)	254 (181–396)	253 (182–376)	254 (180–396)
apoB:AII (mg/L)	4 (2–52)	68 (20–134)**	66 (23–121)**	67 (20–134)*
hCRP (mg/L)	0.026 (0.016–0.20)	0.159 (0.030–1.90)***	0.08 (0.016–5.22)***	0.108 (0.016–5.2)***
apoCIII (mg/L)	28 (27–41)	52 (23–74)*	39 (18–59)	41 (18–74)
apoCIIInB (mg/L)	25 (23–36)	40 (15–59)	31 (14–50)	31 (14–59)
apoB:CIII (mg/L)	3.0 (2.5–8.0)	12 (25–26)*	8 (15–19.0)	10 (15–26)*
LCAT (U/L)	360 (66–450)	237 (125–390)*	180 (66–250)*	207 (66-390)*
CETP (pmol/μL/h)	48 (47–52)	48 (41–62)	49 (41–64)	48 (42–64)
TC/HDL-C	2.54 (1.79–2.67)	4.42 (2.94–7.13)***	3.59 (2.36–6.42)***^†^	4.10 (2.36–7.13)***
LDL-C/HDL-C	1.24 (0.612–1.47)	2.70 (1.57–5.20)***	2.20 (1.11–4.00)***^†^	2.44 (1.11–5.21)***
TG/HDL-C	1.26 (0.52–1.08)	2.94 (1.50–4.64)***	2.35 (1.10–7.14)***^†^	2.50 (1.11–7.14)***
HDL-C/apoAI	0.40 (0.28–0.44)	0.29 (0.20–0.42)***	0.31 (0.24–0.46)***	0.31 (0.20–0.46)***
apoAI/apoB	2.45 (2.25–3.90)	1.74 (0.90–3.36)**	1.96 (1.09–3.15)**	1.86 (0.90–3.36)**
apoAI/apoCIII	57 (42–62)	33 (16–64)**	43 (26–54)**	39 (16–64)**

Values are expressed as median (min–max): * *P* < 0.05, ** *P* < 0.01, *** *P* < 0.001 versus the reference group, ^†^ *P* < 0.05 versus the Tx patients

Spearman’s correlation test (Table 3) for Tx patients with statin therapy showed a significant positive correlation between LCAT and apoCIIInonB and a significant negative correlation between LCAT and TG level, and CETP activity had a significant negative correlation with HDL-C level, HDL-C/apoAI and apoAI/apoB ratios. However, Tx patients without statin showed a significant positive correlation between LCAT and apoB and apoCIII, and CETP activity showed a significant positive correlation with HDL-C/apoAI ratio. All Tx patients showed a significant positive correlation between LCAT and apoB, apoCIII, apoCIIInonB, and hsCRP concentration, and a significant negative correlation with apoAI/apoB and apoAI/apoCIII ratios.

**Table 3:** Spearman’s correlation between LCAT mass and lipids, lipoproteins, hsCRP, and CETP activity in Tx patients with and without statins therapy

	With statin *(n* = 25)		Without statin *(n* = 32)		All Tx *(n* = 57)	
	LCAT	CETP	LCAT	CETP	LCAT	CETP
	*R*		*R*		*R*	
TG	−0.538**	0.035	−0.183	−0.130	−0.176	0.002
HDL-C	0.155	−0.528**	0.059	0.192	−0.113	−0.070
apoB	−0.149	0.240	0.389*	0.062	0.405**	0.096
apoCIII	0.333	0.079	0.357*	0.039	0.529***	−0.054
apoCIIInonB	0.508*	0.241	0.250	0.148	0.426***	0.041
HDL-C/apoAI	0.036	−0.529**	0.054	0.547**	−0.136	0.088
apoAI/apoB	0.041	−0.365*	−0.235	−0.151	−0.305*	−0.179
apoAI/apoCIII	−0.247	−0.057	−0.158	−0.123	−0.474***	−0.023
hsCRP	0.036	−0.097	0.147	0.137	0.275*	0.039

* *P* < 0.05, ** P < 0.01, *** P < 0.001

Multiple ridge stepwise forward regression analysis in all Tx patients (Table 4) showed that LCAT (*R* ^2^ = 0.321) as a dependent variable was associated positively with total apoCIII (*β* = 0.368, *P* = 0.002) and negatively with TG level (*β* = −0.306, *P* = 0.007). The group of selected Tx patients with statin showed a significant negative association between LCAT (*R* ^2^ = 0.261) and TG concentration (*β* = −0.415, *P* = 0.041). However, in Tx patients without statin therapy, LCAT (*R* ^2^ = 0.372) showed a significant positive association with apoAII (*β* = 0.411, *P* = 0.006) and a significant negative association with TG (*β* = −0.492, *P* = 0.002) concentration. Moreover, in Tx patients without statin therapy and with lower TG (114(76–128) and higher HDL-C levels (56.9(43–74.7), LCAT (*R* ^2^ = 0.394) showed a significant positive association with apoAII (*β* = 0.525, *P* = 0.018), a significant positive association with LDL-C (*β* = 0.466, *P* = 0.019), and a significant negative association with apoAI (*β* = −0.449, *P* = 0.039).

**Table 4:** Multiple ridge forward regression between mass of LCAT and lipids, lipoproteins in all Tx patients, and with and without statin therapy, and all Tx patients dichotomized

	All Tx patients *(n* = 57; *R* ^2^ = 0.321)		Tx patients with statin *(n* = 32; *R* ^2^ = 0.261)		Tx patients without statin *(n* = 25; *R* ^2^ = 0.372)		Tx patients without statin *n* = 27 high HDL-C *(R* ^2^ = 0.394)		All Tx patients dichotomized data *(n* = 57; *R* ^2^ = 0.433)	
	*β*	*P*	*β*	*P*	*β*	*P*	*β*	*P*	*β*	*P*
apoAI							−0.449	0.038	−0.249	0.049
apoAII					0.411	0.006	0.525	0.018		
apoAIInB									0.292	0.017
apoB:AII									0.221	0.058
apoCIII	0.368	0.002								
apoCIIInB									0.205	0.056
TG	−0.306	0.007	−0.415	0.041	−0.492	0.002			−0.395	0.0006
LDL-C							0.466	0.019		

Dichotomized multiple ridge stepwise forward regression analysis for all Tx patients showed that LCAT (*R* ^2^ = 0.433) was significantly positively associated with apoAIInonB (*β* = 0.292, *P* = 0.017), significantly negatively associated with apoAI (*β* = −0.249, *P* = 0.049) and TG (*β* = −0.395, *P* = 0.0006), and non-significantly positively associated with apoCIIInonB (*β* = 0.205, *P* = 0.056) and apoB:AII (*β* = 0.221, *P* = 0.058).

## Discussion

Cardiovascular disease is the main cause of mortality in renal transplantation and the second cause of graft loss and death with functioning graft [13]. Reverse kidney renal failure after renal transplantation is associated with various types of metabolic dysfunctions, but lipid and lipoprotein abnormalities appear to progress in Tx patients as a consequence of immunosuppressive therapy [13]. The Tx patients in the present study received cyclosporine A (CSA) (60 %) or prograf (40 %), and additionally MyFortic, Azatioprine, and CelCept. Because these patients had worse lipid profiles, they received statin therapy. However, the Tx patients without statin therapy received prograf (60 %) or cyclosporine A (40 %), and additionally MyFortic, Azatioprine, and CellCept. These patients had better lipid profiles and did not receive anti-lipidemic therapy. The stepwise switch from CSA to MyFortic was safe and mostly successful, and had beneficial effects on blood pressure, glomerular hemodynamics, and lipid profiles. Beneficial trends were already presented after withdrawal of CSA [14].

The in vivo results of the present study showed that Tx patients with and without statin therapy had moderate dyslipidemia, dyslipoproteinemia, and atherogenic lipid and lipoprotein ratios, decreased LCAT mass, and slightly increased hsCRP, but no CETP activity. Spearman’s correlation test between lipids and lipoproteins and LCAT or CETP activity indicated that in Tx patients with statin, moderate hypercholesterolemia, hypertriglyceridemia, and increased TRLs (apoB:CIII and apoB:AII), LCAT was significantly positively correlated with apoCIII and significantly negatively with TG levels. Simultaneously, CETP was significantly negatively correlated with apoAI/apoB, and HDL-C/apoAI and HDL-C composition. In Tx patients without statin, there were lower concentration of TRLs (lower apoB:CIII but not apoB:AII) and beneficial lipid and lipoprotein ratios than Tx patients with statin, but worse than controls; LCAT was significantly positively correlated with apoB and apoCIII. At the same time, CETP was significantly positively correlated with HDL-C/apoAI composition of HDL. In multiple ridge stepwise forward regression, the Tx patients showed that immunosuppressive therapy can cause disturbed lipoprotein composition of HDL and TRLs [15, 16]. Tx patients with higher TG and TRLs levels and disturbed composition of apoCIII, apoAI, apoAII, and TG in HDL can decrease LCAT. However, Tx patients without statin and with lower TG but higher HDL-C and disturbed lipoprotein composition of ApoAI and apoAII in HDL particle can decrease LCAT and increase LDL cholesterol. Moreover, increased TG and LDL-C and hsCRP together with disturbed lipoproteins composition can aggravate renal graft and accelerate atherosclerosis and chronic heart disease. The authors of the present study have shown previously that Tx patients with hypertriglyceridemia have HDL particles remodeled by subclass distribution into smaller-sized particles, that the concentration of HDL particles decreased, and that the HDL-C/apoAI ratio seemed to be a good marker of HDL subclass distribution into smaller size particles [15, 16]. The presented in vivo results of Tx patients suggest that the inhibition or activation of LCAT is due, in part, to HDL-associated lipoprotein. HDL and TRLs composition can contribute to the decrease or increase in LCAT and can modulate all concentrations of TG and/or LDL-C in serum of Tx patients.

Castellani et al. [17] have reported that the function of apoAII is to regulate the metabolism of TRLs, with HDL serving as a pool of apoAII, the same as with the apoC apolipoproteins that is transferred to the TRLs; however, the function of apoAII remains unknown. The re-arrangement in HDL composition, size, and function may be of benefit to the anti-atherogenic HDL particles. LCAT reaction requires not only disorder in the HDL surface, but also specific interaction between the partners [such as apoAI, LCAT, phosphatidylcholine (PCs and cholesterol)] [18]. ApoAII on spherical HDLs: (1) stabilizes the lipoproteins against chemical or thermal denaturation, (2) counteracts the anti-atherogenic effects of apoAI, and (3) decelerates both the denaturation and metabolic remodeling of spherical HDL [18]. Moderate structural disorder accelerates the metabolic remodeling of HDLs, which may benefit the HDL function in cholesterol removal [18]. It has been suggested that apoCIII regulates LCAT activity indirectly through apolipoprotein that activates LCAT [19]. Cho et al. [20] demonstrated that in vitro LCAT activity decreased in response to increased apoCIII content in rHDL and that this occurred in a dose-dependent manner. This decreased ability of rHDL-containing apoCIII to activate LCAT shows that LCAT exhibits 4–5 % LCAT activation ability, while apoAI-rHDL exhibits 100 % reaction rates [21].

Patients with low HDL-C displayed marked changes in their HDL composition and subclass distributions. Larger HDL_2_ particles, as well as HDL mean particle size, are reduced in patients with low HDL-C. Small, dense HDL_3_ particle subclasses have more capacity to protect LDL against oxidation than large, light HDL_2_ particles. Therefore, the low susceptibility of HDL to oxidation in the low HDL-C patients may be due to compositional changes specifically in the HDL_2_ subclass [22]. It has been documented that in hypercholesterolemic patients, LCAT activity was low while CETP activity was high, which was associated with an increased plasma TC level in these patients. The higher the TC levels, the particle size of HDL subclasses has a tendency to be smaller, which suggests that the elevated TC blocked the maturation of HDL subclasses metabolism and impeded the efficiency of RCT. The net effect of CETP action on HDL is depletion of CE and enrichment with TG, with an overall net reduction in the size of the particles [23]. Bailey et al. [24] have provided a biochemical basis for the nascent HDL remodeling pathway that involves plasma apoB-containing lipoprotein and plasma phospholipid transfer protein (PLTP). The free cholesterol content of the model nascent HDL pool was transferred to plasma apoB, which was then redistributed to HDL for esterification by LCAT. Subsequently, CEs were transferred back to plasma apoB by CETP. Simultaneously, PLTP mediated the depletion of the phospholipids content of nascent HDL, leading to the incorporation of lipid-poor apoAI into the plasma resident HDL pool, or conversion to preβ_1_–LpAI.

Bailey et al. also reported that the remodeling of the HDL resident pool by CETP, hepatic triglyceride lipase (H-TGL), and PLTP contributes to the generation of preβ_1_-LpAI or lipid-free apoAI. Importantly, this gives rise to questions regarding the stability and structural integrity of the nascent particles within the plasma environment, the significance of which deserves further investigation [24]. The results of the present study suggest that increased HDL-C does not always protect against cardiovascular disease and can sometimes be associated with increased coronary events [25].

## Conclusion

The results suggest that inhibition or activation of LCAT is due, in part, to HDL-associated lipoprotein. Lipoprotein composition of apoAI, apoAIInonB, and apoCIIInonB in HDL particle and apoB:AII (TRLs) can contribute to decrease LCAT in Tx patients. Tx patients without statin and with lower TG but higher HDL-C, and disturbed lipoprotein composition of ApoAI and apoAII in HDL particle can decrease LCAT, increase LDL cholesterol, aggravate renal graft, and accelerate atherosclerosis and chronic heart disease.
